# Surgical Management of a Traumatic Mainstem Bronchus Avulsion

**DOI:** 10.1016/j.atssr.2024.05.015

**Published:** 2024-06-12

**Authors:** Jonathan B. Livezey, Caitlin E. Jones Sayyid, Daniel L. Miller

**Affiliations:** 1 Department of General Surgery, Dwight D. Eisenhower Army Medical Center, Fort Eisenhower, Georgia; 2 Department of General Surgery, Augusta University Medical Center, Augusta, Georgia; 3 Department of Thoracic Surgery, Augusta University Medical Center, Augusta, Georgia

## Abstract

Traumatic tracheobronchial tree injuries are rarely survivable. We present the case of a 31-year-old male patient who had a delayed discovery of a complete right mainstem bronchus avulsion following a motor vehicle collision. Despite initial respiratory stability, the patient rapidly deteriorated on hospital day 4. Flexible bronchoscopy was performed and demonstrated a right mainstem bronchus avulsion with endobronchial mediastinal adipose tissue partially obstructing and stabilizing the transected airway. The patient successfully underwent a right posterolateral thoracotomy with primary anastomosis of the right mainstem bronchus. High clinical suspicion for tracheobronchial injuries is required after high-speed acceleration-deceleration mechanisms resulting in blunt chest trauma.

Tracheobronchial injuries are severe and often life-threatening if not recognized and managed promptly.^1^ Injury of the tracheobronchial tree can occur due to blunt or penetrating trauma to the neck and thorax. Many of these patients die at the scene, with mortality rates ranging from 30%-80%.^2^ Patients surviving hospital admission often have subcutaneous emphysema, pneumomediastinum, and pneumothoraces with significant air leaks after chest tube placement.^3^ However, due to the nonspecific symptoms and various radiologic signs, delays in diagnosis can occur.^1^

Blunt trauma is an uncommon cause of tracheobronchial injury compared to penetrating trauma and occurs primarily after high-speed motor vehicle collisions.^2^ Considering the rarity of surviving a major blunt tracheobronchial injury, we report this case of an adult male patient who presented with a complete right mainstem bronchus avulsion.

A 31-year-old male individual presented to the emergency department after a motor vehicle rollover with ejection that resulted in cardiac arrest on site. Emergency medical services performed an endotracheal intubation and bilateral needle chest decompressions with return of spontaneous circulation after a single round of cardiopulmonary resuscitation. On arrival to the trauma bay, the patient had diminished breath sounds on the left hemithorax and oxygen saturation greater than 90% on 100% fraction of inspired oxygen. Bilateral large bore chest tubes (36F) were placed with less than 100 mL of blood immediately evacuated and expiratory air leaks only. The patient was hypotensive with faint but palpable distal pulses. Extensive subcutaneous crepitus was noted across his entire torso. This limited the extended focused assessment sonography for trauma (eFAST) exam. Chest radiograph and computed tomography of the chest demonstrated extensive pneumomediastinum and severe subcutaneous emphysema throughout the soft tissues of the neck, chest, and upper abdomen. Due to the patient’s hemodynamic instability with limited eFAST exam, the patient was taken to the operating room for an exploratory laparotomy. This was negative for intraabdominal injury. He was transferred to the surgical trauma intensive care unit for continued resuscitation.

Over the next 3 days, the patient developed a right apical pneumothorax despite appropriate chest tube position and function. His subcutaneous emphysema, pneumomediastinum, and vasopressor requirements sharply increased with immediate concern for obstructive shock secondary to massive air accumulation. Bronchoscopy was performed and demonstrated mediastinal adipose tissue arising from the lateral aspect of the right mainstem bronchus with partial obstruction of the airway consistent with a right mainstem bronchus injury.

The patient was emergently taken to the operating room for a right posterolateral thoracotomy. Upon mediastinal exposure and tracheal dissection, complete avulsion of the right mainstem bronchus just proximal to the right upper lobe takeoff was identified (Figure). The complete avulsion was repaired primarily in an end-to-end fashion. The posterior membrane was approximated with a running 4-0 polydioxanone suture and anterior cartilaginous rings with interrupted 3-0 polydioxanone sutures. A well-vascularized thymic tissue flap was mobilized to buttress the repair in a 360-degree fashion. A chest tube was placed posteriorly with projection to the apex and the thoracotomy closed in layers, which concluded the procedure.

**Figure:**
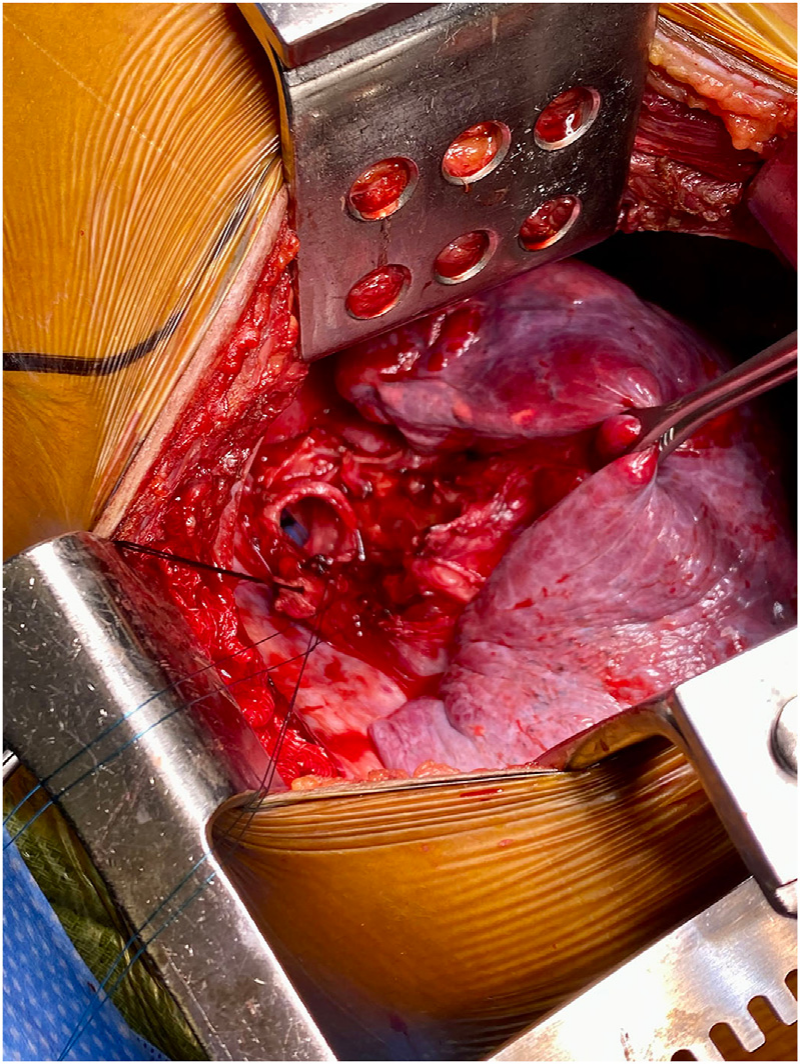
Intraoperative image of a complete avulsion of the right mainstem bronchus just proximal to the right upper lobe takeoff.

On postoperative day 1, a flexible bronchoscopy demonstrated a widely patent right mainstem bronchus anastomosis. The patient continued to recover well with decreasing ventilator requirements and was extubated on postoperative day 3. The patient had a prolonged hospital stay due to his other traumatic injuries and was discharged to a long-term care rehabilitation facility in stable condition with a favorable respiratory and neurologic outcome.

## Comment

Blunt chest trauma resulting in tracheobronchial injury is uncommon and survival of complete avulsion of a mainstem bronchus is rare.^4^, ^5^, ^6^ The most common mechanism is sudden anteroposterior compression, resulting in lateral separation of the carina.^2^

Radiologic imaging and endoscopic examination are crucial for expedited identification of suspected tracheobronchial injuries. Common radiographic signs are subcutaneous emphysema, pneumothorax, and pneumomediastinum. In an upper airway injury, computed tomography imaging may identify paratracheal air, deep cervical emphysema, or pneumomediastinum. In a lower airway injury, computed tomography imaging can demonstrate mediastinal hematoma, a focal intimal flap in the tracheal lumen, or pneumomediastinum. Nevertheless, all patients with a suspected airway injury should undergo early endoscopic examination with laryngoscopy or bronchoscopy. Common findings include tear of the cartilaginous wall, blood clot filling the airway, and collapsed lumen distal to the hematoma. A lesion being traversed by an endotracheal tube can be easily missed and the tube should be carefully retracted during bronchoscopy to ensure complete examination of the airway.^2^ As described in this case, bedside bronchoscopy identified an avulsion injury of the right mainstem bronchus. We believe that mediastinal adipose tissue penetrated through the injury site and stabilized the airway until further positive pressure ventilation and respiratory maneuvers over 3 days extended the injury and reduced the adipose tissue obstruction of the airway, resulting in rapid respiratory collapse.

The goals of operative intervention for tracheobronchial injuries are correcting severe oxygenation and ventilation deficits secondary to massive tidal volume losses and preventing or resolving airway obstruction and mediastinitis.^2^ A primary reconstruction should be attempted if possible. Notably, more than 80% of blunt tracheobronchial injuries occurs within 2.5 cm of the carina.^6^ To have adequate access to the distal third of the trachea, bifurcation, and mainstem bronchi, a right thoracotomy is required. In some situations, a cervical incision or medial sternotomy can also be appropriate, especially if the tracheal injury is above the aortic arch. It is difficult to access the left main bronchus and mobilization of the aorta and division of the ligamentum arteriosum may be required.^2^ A right posterolateral thoracotomy ensured excellent access and exposure to the proximal bronchial injury in our patient.

Injuries to the tracheobronchial tree can be divided based on the axis of the injury (transverse vs longitudinal) and size (greater or less than 2 cm). Small transverse tears can be repaired with interrupted sutures with knots lying outside of the lumen to prevent postoperative irritation and stenosis. Longitudinal lacerations can be repaired with continuous running suture. Complete transection of the tracheobronchial tree requires debridement of devitalized wound edges followed by an end-to-end anastomosis.^2^ This was the repair that was performed in our patient. It is important to avoid excessive airway mobilization as this can lead to devascularization with resulting dehiscence or stenosis. Resection of the tracheobronchial structures should be avoided whenever possible. Using a protective tissue flap of pleura, pericardium, thymic mediastinal fat, or intercostal muscle can be helpful to cover the anastomosis and prevent fistulization to adjacent structures and repair air leaks.^2^

In this report, we described a patient who had complete right mainstem bronchus avulsion requiring emergent right posterolateral thoracotomy with primary anastomosis. Although he recovered well from his repair, the injury was not identified until after he progressed to obstructive shock. Our case demonstrates that maintaining a high clinical suspicion for a tracheobronchial injury is imperative in patients who suffer severe blunt chest trauma from high-speed acceleration-deceleration injuries.
